# Antibacterial Responses by Peritoneal Macrophages Are Enhanced Following Vitamin D Supplementation

**DOI:** 10.1371/journal.pone.0116530

**Published:** 2014-12-30

**Authors:** Justine Bacchetta, Rene F. Chun, Barbara Gales, Joshua J. Zaritsky, Sandrine Leroy, Katherine Wesseling-Perry, Niels Boregaard, Anjay Rastogi, Isidro B. Salusky, Martin Hewison

**Affiliations:** 1 Orthopedic Hospital Research Center, David Geffen School of Medicine at University of California Los Angeles, Los Angeles, California, United States of America; 2 Department of Pediatrics, David Geffen School of Medicine, University of California Los Angeles, Los Angeles, California, United States of America; 3 Centre de Référence des Maladies Rénales Rares, Institut de Génomique Fonctionnelle à l’Ecole Normale Supérieure de Lyon et Université de Lyon, Lyon, France; 4 Unité d’épidémiologie des maladies émergentes, Institut Pasteur, Paris, France; 5 Department of Medicine, David Geffen School of Medicine, University of California Los Angeles, Los Angeles, California, United States of America; 6 Department of Hematology, University of Copenhagen, Copenhagen, Denmark; Cardiff University School of Medicine, United Kingdom

## Abstract

Patients with chronic kidney disease (CKD), who usually display low serum 25-hydroxyvitamin D (25D) and 1,25-dihydroxyvitamin D (1,25D), are at high risk of infection, notably those undergoing peritoneal dialysis (PD). We hypothesized that peritoneal macrophages from PD patients are an important target for vitamin D-induced antibacterial activity. Dialysate effluent fluid was obtained from 27 non-infected PD patients. Flow cytometry indicated that PD cells were mainly monocytic (37.9±17.7% cells CD14^+^/CD45^+^). Ex vivo analyses showed that PD cells treated with 25D (100 nM, 6 hrs) or 1,25D (5 nM, 6 hrs) induced mRNA for antibacterial cathelicidin (*CAMP*) but conversely suppressed mRNA for hepcidin (*HAMP*). PD cells from patients with peritonitis (n = 3) showed higher baseline expression of *CAMP* (18-fold±9, p<0.05) and *HAMP* (64-fold±7) relative to cells from non-infected patients. In 12 non-infected PD patients, oral supplementation with a single dose of vitamin D_2_ (100,000 IU) increased serum levels of 25D from 18±8 to 41±15 ng/ml (p = 0.002). This had no significant effect on PD cell CD14/CD45 expression, but mRNA for *HAMP* was suppressed significantly (0.5-fold, p = 0.04). Adjustment for PD cell CD14/CD45 expression using a mixed linear statistical model also revealed increased expression of *CAMP* (mRNA in PD cells and protein in effluent) in vitamin D-supplemented patients. These data show for the first time that vitamin D supplementation in vitro and in vivo promotes innate immune responses that may enhance macrophage antibacterial responses in patients undergoing PD. This highlights a potentially important function for vitamin D in preventing infection-related complications in CKD.

## Introduction

In patients with chronic kidney disease (CKD), vitamin D-deficiency is a persistent problem [1], [2]. Current guidelines for the management of adult and pediatric CKD alterations of bone and mineral metabolism recommend target levels for 25-hydroxyvitamin D (25D), the major circulating form of vitamin D, of at least 20 ng/mL (50 nM) [3]–[5]. Despite this, vitamin D deficiency remains common in CKD patients [6], most notably in pediatric patients where a 40–80% prevalence of low serum 25D has been reported [7]–[12]. Previously the management of vitamin D-deficiency in CKD was focused on the use of active 1,25-dihydroxyvitamin D (1,25D) to control secondary hyperparathyroidism and associated skeletal/calciotropic dysfunction [13], although supplementation with vitamin D itself has been shown to delay the onset of secondary hyperparathyroidism [10]. Other studies highlighting diverse effects of vitamin D on cardiovascular [14], [15] and immune function [16], [17] support broader benefits of vitamin D supplementation in CKD patients [18]. Conversely, vitamin D-deficiency may impair key extra-renal responses to vitamin D, notably innate immune responses to infection [19], [20].

Patients with CKD are at high risk of infection [21], notably those undergoing peritoneal dialysis (PD) [22]–[24]. Immune responses in the peritoneum are of immediate relevance to PD patients because of their close link with important morbidities such as peritonitis and the increased risk of further treatment failure [25]. The peritoneum has abundant cells capable of supporting immune response to peritoneal infection [26], with the predominant cell type being macrophage-like [27]–[29]. Macrophages are key target cells for vitamin D, with intracrine expression of the enzyme 1α-hydroxylase (*CYP27B1*) catalyzing local synthesis of 1,25D, which is then able to transcriptionally regulate key antimicrobial proteins such as cathelicidin (*CAMP*), as well as classical targets such as its catabolic enzyme 24-hydroxylase (CYP24A1), via the nuclear vitamin D receptor (VDR) [30]. Recent studies have shown that 25D and 1,25D also act to decrease expression of another antibacterial protein, hepcidin (*HAMP*), resulting in increased expression of ferroportin, the only known exporter of intracellular iron [31]. This suggests an alternative antibacterial function for vitamin D involving suppression of intracellular iron concentrations. Based on these observations we postulated that vitamin D (25D)-deficiency associated with CKD may predispose to infection through impaired regulation of *CAMP* and *HAMP* activity.

In a case-control study of adult patients undergoing hemodialysis, low serum levels of cathelicidin protein (hCAP) was an independent risk factor for death due to infection, with serum hCAP correlating with circulating 1,25D but not 25D [32]. Paradoxically, other studies have shown that vitamin D therapy decreased expression of *CAMP* in peripheral blood mononuclear cells [33]. In both cases the apparent lack of 25D-mediated induction of hCAP/*CAMP* was attributed to the absence of patient infection and associated induction of *CYP27B1*. However, it seems unlikely that intracrine induction of innate immunity by 25D will be manifested by changes in circulating levels of antibacterial proteins, but will instead reflect localized effects on tissue macrophages at sites of infection. With this in mind, we hypothesized that peritoneal cells may provide a more sensitive indicator of innate immune responses to altered 25D status in PD patients. These cells are not routinely used to assess immune function because protocols for isolating cells from dialysate effluent are time consuming [34]–[38]. Nevertheless, previous studies have shown that PD cells express a functional system for synthesis of 1,25D [39], with this activity being greatly enhanced in cells from patients with peritonitis [39], [40]. We therefore investigated the capacity for vitamin D-mediated intracrine regulation of antibacterial *CAMP* and *HAMP* in PD cells ex vivo and in vivo. Activation of such a mechanism may enhance innate immune activity in CKD patients and thus help to prevent infection and associated morbidities.

## Results

### Clinical characteristics, serum biochemistry, and PD cell phenotype/gene expression in dialyzed patients

Baseline data for all the patients studied are shown in **Table 1** (this includes the first baseline sample for the subset of patients who subsequently participated in the vitamin D supplementation trial), and the underlying renal diseases associated with the patient cohort are summarized in Table S1 in **S1 File**. Serum concentrations of 25D were 18±8 ng/ml. Flow cytometry indicated that PD cells were mainly monocytic/macrophage-like, with 37.9±17.7% being CD14^+^/CD45^+^, while 25.4±14.5% were CD14^−^/CD45^+^ and 32.1±19.9% were CD14^−^/CD45^−^. Linear regression analyses using baseline samples showed that there was no significant correlation between PD cell expression of CD14 and CD45 and patient serum 25D levels (data not shown). However, there was an inverse correlation between PD cell *CYP27B1* and *VDR* mRNA, and the number of CD14^−^/CD45^+^ cells (R = 0.429, p = 0.014 and R = 0.358, p = 0.037 respectively) (**Figures S1A and 1C in** **S2 File**). Collectively these data suggest that expression of the intracrine vitamin D system is linked to CD14 expression.

**Table 1:** Clinical and biological data for PD patients at baseline.

**Patients**	**Number of patients (pediatric/adults)**	27 (19/8)
	**Age (years)**	18.1 (2.7–39.1)
	**Number of males (pediatric/adults)**	16 (12/4)
	**Dialysis duration (years)**	2.7 (0.1–12.1)
	**KT/V at the time of evaluation**	2.1±0.9
**Baseline biological data**	**Serum calcium (mg/dL)**	8.8±1.0
	**Serum phosphorus (mg/dL)**	5.1±0.9
	**Serum PTH (pg/mL)**	418 (24–1571)
	**Serum 25D (ng/mL)**	18 (8–55)
	**Serum total alkaline phosphatase (IU/L)**	148 (57–788)
	**Serum albumin (g/L)**	42±5
	**Serum hemoglobin (g/dL)**	11.9±2.2
	**Serum BUN (mg/dL)**	45.4±16.4
	**Serum ferritin (µg/L)**	314 (35–1106)
	**Plasma FGF23 (RU/mL)**	2902 (307–42505)
	**Peritoneal dialysate FGF23 (RU/mL)**	69 (11–472)
	**Peritoneal dialysate hCAP (pg/ml)**	2.2 (0.4–7.7)
**PD cell analyses**	**CD14+/CD45+ monocyte/macrophage (% of cells)**	38±18
	**CD14−/CD45− non-leukocyte (% of cells)**	32±20
	**CD45+/CD14− non-monocytic (% of cells)**	25±15
	***CYP27B1*** ** (ΔCt)**	16.2±1.6
	***CYP24A1*** ** (ΔCt)**	20.0±2.9
	***VDR*** ** (ΔCt)**	14.7 (11.2–20.6)
	***CAMP*** ** (ΔCt)**	22.1±1.5
	***HAMP*** ** (ΔCt)**	17.8±1.6

Expression or lack of expression of CD14 and CD45 is shown as % of CD14^−/+^ or CD45^−/+^ PD cells. Expression of mRNAs for *CYP27B1*, *CYP24A1*, *VDR*, *CAMP*, and *HAMP*, are shown as ΔCt values relative to expression of the housekeeping gene 18S rRNA, the lower the ΔCt the higher the expression of the gene of interest. Data are presented as mean ± SD for variables with normal distributions, or median (range) for variables with skewed distribution.

**Figure 1:**
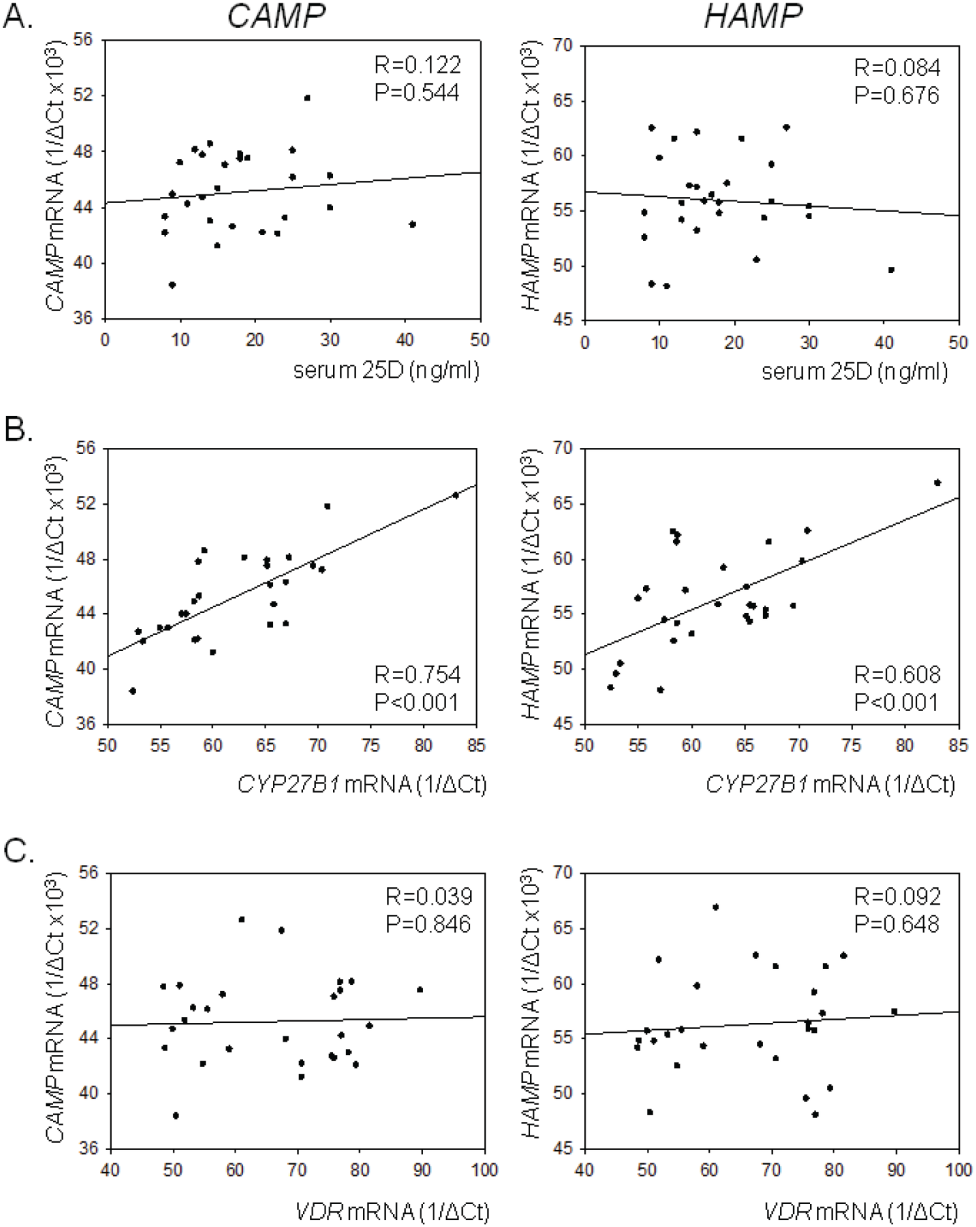
Vitamin D-related gene expression in CKD patient PD cells at baseline. Correlation between PD cell mRNA expression of *CAMP* or *HAMP* and: A) donor patient serum concentrations of 25D (ng/ml); B) *CYP27B1* mRNA; C) *VDR* mRNA in PD cells isolated from CKD patient dialysate effluents from n = 27 baseline samples (pre-vitamin D supplementation) from 27 different patients. Data for mRNA expression are shown as 1/ΔCt values.

Baseline data were further analyzed to assess the relationship between PD cell type, serum 25D, and the intracrine vitamin D system on three markers of PD cell immune function: 1) PD cell mRNA for *CAMP*; 2) PD cell mRNA for *HAMP*; 3) hCAP protein concentrations in PD dialysate effluent. There was no significant correlation between PD cell CD14/CD45 expression and any of these markers (data not shown), and no significant correlation between baseline serum concentrations of 25D and PD cell mRNA for *CAMP* or *HAMP* (**Fig. 1A**). However, PD cell mRNA for *CYP27B1* correlated with both *CAMP* (R = 0.737, P<0.001), and *HAMP* (R = 0.544, P = 0.047) (**Fig. 1B**). By contrast, PD cell *VDR* did not correlate with *CAMP* or *HAMP* **(** **Fig. 1C**). Similar analysis of hCAP concentrations in dialysate effluent showed no correlation with PD cell type or any of the PD cell mRNAs analyzed in **Fig. 1** (data not shown).

### Effects of vitamin D metabolites on PD cell gene expression *ex vivo*

**Figure 2:**
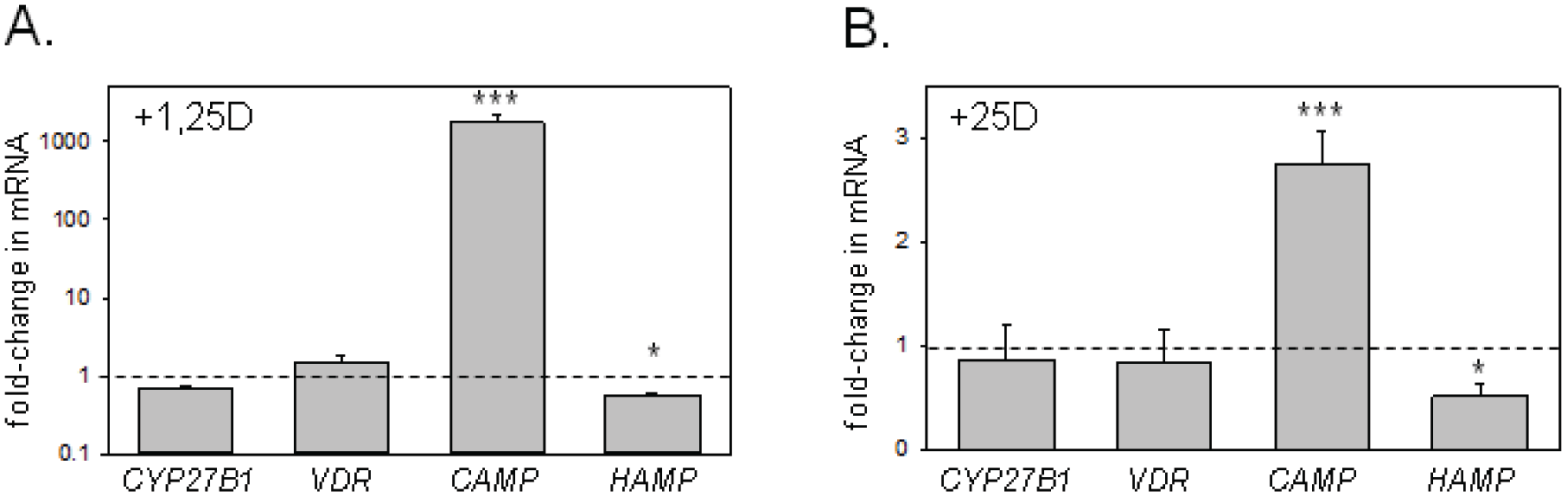
Effect of 25D and 1,25D on vitamin D-related gene expression in baseline PD cells in vitro. Effect of 1,25D (5 nM, 6 hrs, n = 13) (A) or 25D (100 nM, 6 hrs, n = 8) (B) on expression for mRNA for *CYP27B1*, *VDR*, *CAMP* and *HAMP* in PD cells cultured in RPMI with 10% human serum and GM-CSF (10 IU/mL). Data are shown as fold-change in mRNA expression relative to vehicle (0.1% ethanol)-treated cells for each gene with vehicle expression = 1 (see dashed line). * = statistically different from vehicle-treated cells, p<0.05. *** = statistically different from vehicle-treated cells, p<0.001.

Ex vivo treatment of PD cells from non-infected patients with 25D or 1,25D increased expression of *CAMP*, with 1,25D (1729-fold, p<0.001) (**Fig. 2A**) being more potent than 25D (2.81-fold, p<0.001) (**Fig. 2B**). By contrast, both 1,25D and 25D suppressed expression of *HAMP* to a similar extent (0.57-fold, p<0.05 and 0.52-fold, p<0.05 respectively) (**Fig. 2A and 2B**).

### Analysis of PD cells from a subset of patients with peritonitis

**Figure 3:**
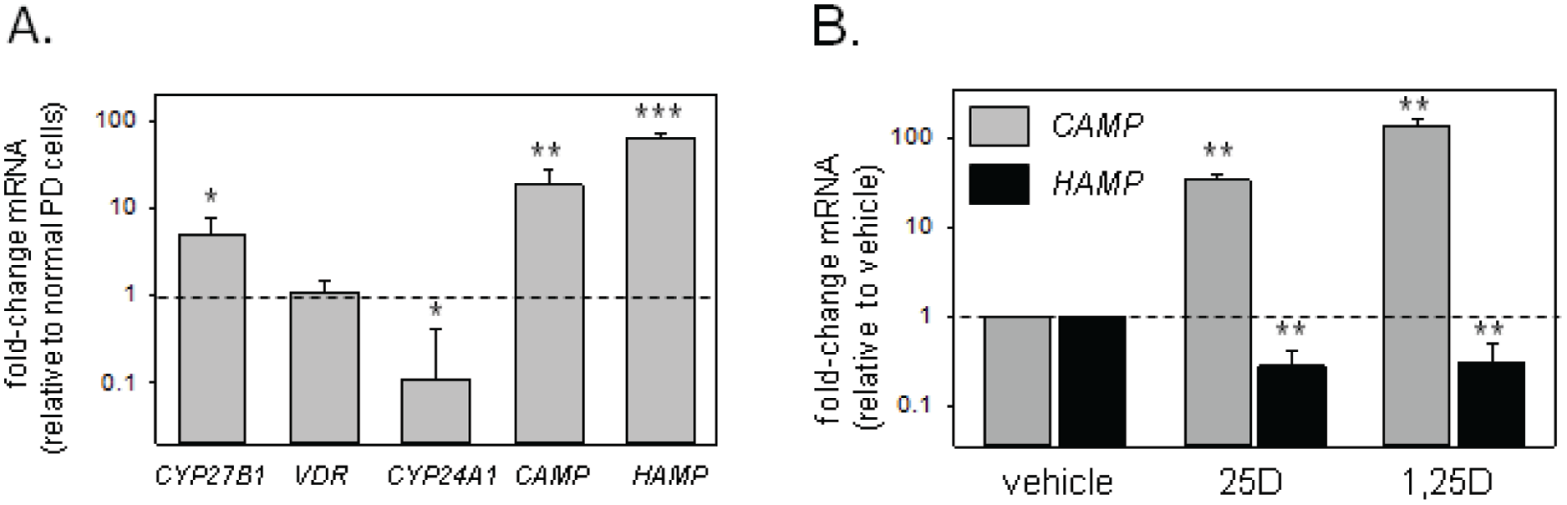
Baseline expression, and 25D/1,25D-regulation of vitamin D-related genes in PD cells from patients with peritonitis. (A) Relative expression of mRNA for *CYP27B1*, *VDR*, *CYP24A1*, *CAMP* and *HAMP* in PD cells from peritonitis patients (n = 3) compared to PD cells from non-infected patients (n = 8). Data are shown as fold-change in mRNA expression relative to non-infected cells. (B) Effect of 1,25D (5 nM, 6hrs) or 25D (100 nM, 6 hrs) on expression for mRNA for *CAMP* (grey bars) or *HAMP* (black bars) in PD cells from peritonitis patients (n = 3) RPMI with 10% human serum and GM-CSF (10 IU/mL). Data are shown as fold-change in mRNA expression relative to non-infected PD cells (3A) or vehicle (0.1% ethanol)-treated cells (3B). * = statistically different from vehicle-treated (0.1% ethanol) cells, p<0.05. ** = statistically different from vehicle-treated cells, p<0.01.

PD cells from patients with peritonitis (n = 3) showed increased expression of *CYP27B1* relative to PD cells from non-infected patients (4.97-fold higher±2.75, p<0.01) (**Fig. 3A**). There was no significant difference in *VDR* expression between the two patient groups, but the catabolic enzyme *CYP24A1* was lower in PD cells from peritonitis patients (0.11-fold±0.31, p<0.05). Expression of both *CAMP* (18.3-fold±9.4, p<0.01), and *HAMP* (64.1-fold±7.0, p<0.001) was increased in peritonitis PD cells from compared to non-infected patients. Elevated hCAP protein was also observed in dialysate effluent. This measurement was only available for two peritonitis samples but, in contrast to the low levels of hCAP observed in dialysate from non-infected patients (median 2.24, range 0.37–7.67 pg/mL), the acute peritonitis samples showed 12.6 and 131.8 pg/mL hCAP respectively. Data in **Fig. 3B** showed that *CAMP* expression in peritonitis PD cells was further elevated following ex vivo treatment with 1,25D (135.3-fold±24.9, p<0.01) or 25D (34.2-fold±5.2, p<0.01). For *HAMP*, 25D or 1,25D treatments were equally effective in suppressing expression (0.28-fold±0.12, p<0.01 and 0.31-fold±0.30, p<0.01 respectively).

### Effect of vitamin D supplementation *in vivo* on PD cell phenotype and gene expression

To explore vitamin D and PD cell immune function in vivo, further studies were carried out using dialysate effluent samples from twelve patients who completed a pilot vitamin D supplementation trial, in which serum levels of 25D in baseline sample 1 (B1) and B2 respectively increased from 17.5 (13.5–24.5) ng/ml, and 16.0 (10.0–23.5) ng/ml, to 41.5 (27.5–43.0) ng/ml (p = 0.002) (**Table 2**), with a one month period between B1 and B2. Serum PTH and FGF23 were unchanged from baseline following vitamin D supplementation, and there was no change in PD cell CD14/CD45 expression. Basic statistical analysis also revealed no significant effect of vitamin D supplementation on PD cell mRNA for *CAMP*, but PD cells showed lower levels of *HAMP* (0.5-fold, p = 0.002) following vitamin D supplementation. To account for variations in cells expressing CD14/CD45 in each sample, further statistical analysis of *CAMP* and hCAP expression was carried out using Mixed Linear Modeling. After adjustment for CD14^−^/CD45^+^ cells, there was a significant increase in PD cell expression of *CAMP* following vitamin D supplementation (1.85-fold, P = 0.03) (Table S2 in **S1 File**). Additional adjustment for age yielded a similar result (Table S2 in **S1 File**). Adjustment for numbers of CD14^−^/CD45^+^ or CD14^+^/CD45^+^ cells also revealed an increase in the concentrations of dialysate hCAP protein following vitamin D supplementation (p = 0.02 for both models) (Table S3 in **S1 File**).

**Table 2:** Serum biochemistry, PD cell flow cytometry and PD cell gene expression in CKD patients before and after supplementation with vitamin D_2_.

	Variable	Time point	Median (IQR)	Comparison between baseline samples, p*	Comparison between first baseline and post- treatment samples, p*
**Flow cytometry**	**CD14–**	B1 (**−**1 month)	24.6 (16.8–38.5)	0.3	0.2
	**CD45+ (% of cells)**	B2 (0 month)	20.7 (12.9–23.7)		
	**non-monocytic**	TT (+1 month)	15.2 (4.7–19.8)		
	**CD14+**	B1	38.6 (19.2–52.7)	**0.01**	0.2
	**CD45+ (% of cells)**	B2	53.2 (24.8–58.8)		
	**monocyte/macrophage**	TT	61.2 (24.7–81.9)		
	**CD14−**	B1	23.4 (15.4–54.1)	0.2	1.0
	**CD45− (% of cells) non-leukocyte**	B2	21.2 (16.4–24.3)		
		TT	19.3 (10.5–38.1)		
**mRNA expression (ΔCt)**	***CYP24A1*** ** (ΔCt)**	B1	20.4 (18.2–21.9)	0.4	0.8
		B2	20.0 (18.5–22.2)		
		TT	21.6 (18.8–22.1)		
	***CYP27B1*** ** (ΔCt)**	B1	17.1 (16.8–18.0)	0.1	0.8
		B2	17.3 (16.9–18.5)		
		TT	17.6 (16.4–18.4)		
	***VDR*** ** (ΔCt)**	B1	13.3 (12.5–17.0)	0.4	0.8
		B2	13.2 (12.7–17.6)		
		TT	13.5 (12.2–16.8)		
	***LL37*** ** (ΔCt)**	B1	23.0 (22.6–24.0)	0.4	0.4
		B2	23.3 (22.4–23.7)		
		TT	23.1 (20.9–23.5)		
	***HAMP*** ** (ΔCt)**	B1	17.9 (16.9–19.3)	0.5	**0.04 (0.5-fold decrease)**
		B2	18.2 (17.5–20.0)		
		TT	18.9 (17.4–19.6)		
**Blood markers**	**FGF 23 (RU/mL)**	B1	3302 (1812–10511)	0.3	**0.08**
		B2	2982 (1753–6362)		
		TT	1957 (1317–9461)		
	**PTH (pg/mL)**	B1	201 (41–699)	0.9	0.4
		B2	190 (54–486)		
		TT	276(38–447)		
	**25D (ng/mL)**	B1	17.5 (13.5–24.5)	0.8	**0.002 (2.37-fold increase)**
		B2	16.0 (10.0–23.5)		
		TT	41.5 (27.5–43.0)		
**Dialysate**	**hCAP (pg/ml)**	B1	2.8 (1.9–6.4)	0.5	1.0
		B2	3.0 (1.4–7.9)		
		TT	3.6 (1.5–6.0)		
	**FGF23 (RU/mL)**	B1	86.5 (34.5–120.5)	0.2	0.2
		B2	53.0 (28.5–76.5)		
		TT	60.5 (29.0–107.5)		

Expression or lack of expression of CD14 and CD45 is shown as % of CD14^−/+^ or CD45^−/+^ PD cells. Expression of mRNAs for *CYP27B1*, *CYP24A1*, *VDR, CAMP* and *HAMP*, are shown as ΔCt values relative to housekeeping gene 18S rRNA, the lower the ΔCt the higher the expression of the gene of interest. Time points: Baseline 1 (B1, −1 month), baseline 2 (B2, 0 month), post-treatment (TT, +1 month). Results show median (inter-quartile range) values. *A Kruskall Wallis non parametric test was used to compare baseline samples, and baseline 1 with post- treatment samples.

## Discussion

Intracrine conversion of 25D to active 1,25D appears to be a pivotal mechanism in vitamin D-mediated regulation of monocyte/macrophage antibacterial activity [20], with this response being compromised under conditions of vitamin D-deficiency [30], but enhanced upon restoration of serum 25D levels following vitamin D supplementation [41]. Intracrine innate immune responses to 25D include enhanced autophagy [42], and induction of antibacterial proteins such as cathelicidin [30] and β-defensin 2 [43]. More recently we have shown that 25D can also promote innate immune activity in macrophages by suppressing expression of another antibacterial protein, hepcidin, encoded by the *HAMP* gene [31]. Expression of *HAMP* mRNA was 60-times higher in PD cells from peritonitis patients relative to uninfected patients. This is consistent with previously reviewed responses to infection [44], and may be part of a program of peritoneal antibacterial responses, with *CAMP* also being induced (18-fold) in PD cells from patients with peritonitis. Furthermore, in the two peritonitis samples available for measuring concentrations of hCAP protein in dialysate effluent, hCAP concentrations were also elevated relative to uninfected patients, suggesting that PD macrophages are the source of the dialysate hCAP. Although the data from peritonitis samples are promising, it will be important in future studies to carry out prospective analyses using larger cohorts of infected peritoneal dialysis patients.

The key function of hepcidin appears to be the suppression of cell membrane expression of ferroportin, the only known exporter of intracellular iron [45]. The hepcidin-ferroportin axis in cells such as enterocytes, hepatocytes and macrophages plays a key role in the hypoferremia of infection [46]. Pathogens such as bacteria utilize iron to maintain growth, and thus restriction of circulating iron concentrations represents an important host response to systemic infection [47]. Conversely, intracellular accumulation of iron can promote bacterial growth within cells, and thus additional mechanisms may be required to combat intracellular infection. Data presented in the current study strongly support a role for vitamin D as a sensitive regulator of hepcidin in peritoneal macrophages, with this effect complementing more established induction of antibacterial *CAMP*/hCAP.

There was no significant correlation between baseline patient serum 25D levels and PD cell *CAMP* or *HAMP*. This is possibly due to the low circulating levels of 25D in this population (<20 ng/ml), but could also be influenced by expression of the machinery for 25D activation and 1,25D signaling. At baseline, PD cell *CAMP* and *HAMP* correlated significantly with expression of the 25D-activating enzyme *CYP27B1* (**Fig. 1**). Baseline variations in the vitamin D system may be due to intra-individual exposure to immune stimulators capable of activating pathogen-sensing mechanisms such as the TLR system [48]. This is endorsed by the increased expression of *CYP27B1* and decreased expression of *CYP24A1* in PD cells from peritonitis patients. In PD cells from non-infected patients *CYP27B1* expression may also be influenced by factors associated with CKD. Plasma levels of FGF23 correlated strongly with the FGF23 in dialysate effluents (Figure S2 in **S2 File**), but there was no correlation between FGF23 and either its known target, *CYP27B1*, or immune markers such as *HAMP* and *CAMP*. This may be due to the relatively low levels of *CYP27B1* and *CAMP* expression in PD cells from non-infected patients, and it will be interesting in future studies to assess the possible impact of FGF23 on vitamin D-mediated immune responses in patients with peritonitis.

It is also important to recognize that some patients with end stage renal disease active vitamin D (calcitriol or synthetic vitamin D analog) therapy to help control secondary hyperparathyroidism. In the current study, 8 of the 12 patients who took part in the vitamin D supplementation pilot study were receiving calcitriol/analog therapy. Although the numbers of patients not receiving calcitriol/analog therapy was low, analysis of the two groups of patients suggested that this had no effect on the efficacy of vitamin D supplementation in raising serum concentrations of 25D (Figure S3A in **S2 File**). However, intriguingly, patients who did not receive calcitriol/analog therapy showed a stronger suppression of hepcidin relative to treated patients (Figure S3B in **S2 File**). A possible mechanism for this is unclear as there did not appear to be any significant difference between the calctriol/analog-treated and untreated groups for expression of components of the vitamin D system (data not shown). Similarly, stratifying the patients according to calcitriol/analog therapy did not appear to have any impact on expression of *CAMP* (data not shown). However, although the numbers of patients in the therapy groups is small, these data suggest that effects of vitamin D supplementation, and raised serum 25D, occur irrespective of underlying therapy with active vitamin D. This provides further support for an intracrine model of vitamin D-mediated immunity involving localized synthesis of 1,25D, and will be a key feature of future studies of vitamin D supplementation in kidney disease.

Other variables that may also influence the immune impact of vitamin D supplementation in dialysis patients include the underlying cause of the renal disease, and residual renal function. Although we were unable to show any significant effect of these parameters on patient responses following vitamin D supplementation, residual renal function did show some associations for baseline data (Table S4 in **S1 File**). As expected, patients with residual renal function had decreased circulating concentrations of FGF23 and lower serum levels of creatinine. They were also significantly younger, were on dialysis for less time, had decreased PD monocyte expression of *CYP24A1* mRNA, but had higher PD monocyte mRNA for *CAMP*. This potential link between residual renal function, and attenuated peritoneal monocyte catabolism of vitamin D and enhanced antibacterial expression will be another important target for future studies.

Inter-patient variations in PD cell expression of the vitamin D system may also be due to the composition of cells present in the peritoneum during dialysis. Both CD14^+^ macrophages and CD14^−^ neutrophils express *CAMP* [49], [50] but, unlike macrophages, neutrophils do not appear to synthesize 1,25D. As such these cells are unlikely to be affected by changes in 25D concentration but will nevertheless contribute to PD cell expression of *CAMP*, with this effect varying depending on individual dialysate neutrophil levels. It was therefore interesting to note that following supplementation with vitamin D in vivo, a significant increase in PD cell expression of *CAMP* was only observed following adjustment for CD14^−^ expression. This contrasts the in vivo vitamin D suppression of PD cell *HAMP* without adjustment for cell phenotype, which can be explained by the predominant expression of this gene in *CYP27B1*-expressing macrophages. In the context of monocyte expression of *HAMP*, it was interesting to note that dual expression of the monocyte marker CD14 and common leukocyte antigen CD45 was statistically different between baseline samples 1 and 2 (**Table 2**). This underlines the wide inter-patient variations in peritoneal immune cells, but also further endorses the suppression of *HAMP* expression, relative to baseline sample 1, following vitamin D supplementation. In essence, the apparent higher numbers of monocytic (CD14^+^/CD45^+^), *HAMP*-expressing, cells in B2 would enhance the possibility of vitamin D-mediated suppression of *HAMP*. The fact that HAMP expression was suppressed by vitamin D supplementation without any adjustment for immune cell population highlights the importance of this antibacterial pathway for vitamin D, particularly in the context of kidney disease. The ability of PD cells to mount an adequate antibacterial response to infection may therefore be dependent on their immune composition as well as the local concentration of 25D. In this regard it was interesting to note that analysis of the dialysate effluent samples did not reveal any detectable 25D (data not shown). This suggests that exposure to elevated concentrations of 25D occurs in the general circulation, with the resulting monocytes migrating across the peritoneal membrane into the peritoneal cavity.

Previous studies primarily focused on ex vivo analyses have hypothesized a role for vitamin D in promoting antibacterial immune responses. By analyzing in vivo responses to supplementation with vitamin D, the current study supports this hypothesis, whilst also highlighting some of the limitations of ex vivo analyses. Data suggest that PD cells are an important target for vitamin D, with enhanced 25D promoting antibacterial *CAMP* and suppressing iron-regulatory *HAMP*. PD cells show similar expression of the vitamin D intracrine sytem (*CYP27B1*/*VDR*) relative to peripheral blood mononuclear cells [51]. It therefore seems likely that responses to 25D in vivo will be highly efficient at barrier sites such as the peritoneum where macrophages make up a greater proportion of the total cell population, and where they are more likely to encounter a pathogen. In this setting, despite no apparent infection, patients may nevertheless demonstrate enhanced antibacterial responses when serum 25D levels are elevated and this may help to prevent more widespread infection in PD patients. Less clear is whether vitamin D status or supplementation will have an impact on innate immune responses following peritoneal infection. Further clinical studies are required to determine the extent to which acute vitamin D supplementation and/or therapeutic vitamin D analogs may help to prevent and/or treat infection associated with CKD.

## Materials and Methods

### Patients

We recruited children and young adults (below 40 years of age) undergoing PD, and followed in the Davita Dialysis Center at UCLA, to participate in a study to collect PD effluents and assess circulating markers of bone and mineral metabolism, PD cell phenotype and gene expression. A pilot vitamin D supplementation trial was also carried out in a subset of patients. The project was approved by the UCLA ethic committee, referred to as the Institutional Review Board (ethics approval #10-07-003-01). Patients and their parents/caregivers gave written consent before enrollment. Original copies of the written consent are stored within the administrative offices of the Division of Pediatric Nephrology at the David Geffen School of Medicine at UCLA.

Baseline data were obtained in 19 children and 8 young adults. Patients were treated with continuous cycling peritoneal dialysis using a standard double-bag dialysate system, with 1.36% glucose dialysate. All patients had been free of peritonitis and growth hormone therapy for at least 3 months prior to sample collection. In addition they were not receiving, antibiotics, corticosteroids or other immunosuppressing medications at the time of sample collection. Three separate patients with acute peritonitis episodes were studied at baseline to evaluate the impact of acute infections on the main parameters assessed in this study. Of the 19 children and 8 young adults who were assessed at baseline, 7 children and 5 adults successfully completed a pilot study to assess the impact of oral vitamin D supplementation. Patients above 25 kg received 50,000 IU vitamin D_2_ twice a week for 4 weeks, and those below 25 kg weight received 50,000 IU once a week for 4 weeks. Patients served as their own controls: for each patient, two serum and PD dialysate samples were obtained before vitamin D supplementation (Month −1, Month 0), and a third sample was then obtained after one month of vitamin D_2_ therapy. Circulating levels of 25D, PTH, and FGF23 were measured with an RIA kit, a first-generation immunometric assay (Immutopics San Clemente, CA, normal range 10–65 pg/mL) and a 2^nd^ generation C-terminal kit (Immutopics San Clemente, CA), respectively. Concentrations of hCAP in dialysate effluent were measured with a specific ELISA as described previously [52].

### Isolation of PD cells from dialysate effluent

Peritoneal dialysate effluents were collected using previously described protocols [51]. Briefly, an initial dialysate aliquot (10 mL) was stored at −80°C for measurement of FGF23 and hCAP. For isolation of PD cells, total overnight dialysate effluents were decanted into 500 ml sterile centrifuge tubes, and then centrifuged at 1200G for 10 mins at room temperature. Supernatants were discarded and cell pellets were combined and re-centrifuged using the same parameters. Viable cells were counted in a haemocytometer after staining with Trypan blue. After a third centrifugation remaining cells were either: 1) treated with RNAzol and frozen at −80°C; 2) prepared for flow cytometric analysis; 3) used for ex vivo culture.

### Flow cytometry

Monoclonal antibodies against CD45 (common leukocyte antigen) and CD14 (monocyte marker) conjugated with FITC and R-PE (MHCD-4501 and MHCD-1404) respectively (Invitrogen Corporation, Camarillo, CA, USA) were used for flow cytometric analysis of immune cell phenotype of PD cells. Cells were analyzed using a FACS Caliber flow cytometer (BD LSRII and FACS-DIVA software, Becton-Dickinson, Franklin Lakes, NJ, USA).

### Culture of PD macrophages

PD cells were seeded at 1–5×10^6^ cells/mL in medium containing RPMI 1640, 10% human serum (HS, Human AB serum, Omega Scientific, Tarzana, CA, USA) in 12-well plastic culture plates. After 12–15 hrs of culture to ensure attachment of monocytes, cells were washed once with medium and then re-incubated in RPMI with 10% HS and GM-CSF 10 IU/mL (PeproTech Inc., Rocky Hood, NJ, USA). Treatments were carried out using 25D (100 nM), 1,25D (5 nM), or vehicle (ethanol 0.1%), for 6 hrs, at 37°C in 5% CO_2_. After incubation cells were lysed with RNAzol and RNA analyzed by quantitative RT-PCR (qRT-PCR) as described below.

### Extraction of RNA and reverse transcription

RNA was extracted as described previously [51]. Aliquots (300 ng) of RNA in RNase-free water were reverse-transcribed as recommended by the manufacturer (SuperScript III Reverse Transcriptase, Invitrogen, Carlsbad, CA), and as previously described [51].

### qRT-PCR amplification of cDNAs

Expression of mRNAs for *VDR*, *CYP27B1*, 24-hydroxylase (*CYP24A1*), *CAMP* and *HAMP* was quantified as previously described [51]. Approximately 7.5 ng of cDNA was used per reaction. All reactions were normalized by multiplex analysis with the housekeeping 18S rRNA gene (Applied Biosystems, Foster City, CA, USA). Data were obtained as Ct values (cycle number at which logarithmic PCR plots cross a calculated threshold line). ΔCt values, corresponding to the difference between the Ct of the target gene and the Ct of the housekeeping 18S rRNA gene, were then calculated. PCR amplification of cDNA was conducted using Taqman gene expression assays, as previously described [51]. Probe and primer sets were as follows: Hs00172113-m1 (*VDR)*, Hs00168017-m1 (*CYP27B1)*, Hs00167999-m1 (*CYP24A1)*, Hs00189038-m1 (*CAMP)* and Hs00221783_m1 (*HAMP)* (Applied Biosystems). All reactions were amplified under the following conditions: 95°C for 10 mins, 40 cycles of 95°C for 30 seconds, 55°C for 1 min and 72°C for 1 min. Reactions were initially expressed as mean ± SD ΔCt values and these data were used for all statistical analyses. Values for fold-change relative to vehicle-treated cells were determined using the equation 2^− ΔCt^. For correlation analyses, data were also shown as 1/ΔCt values to more conveniently represent mRNA expression.

### Statistical analysis

Data are presented as mean±SD for variables with normal distributions, or median (range) for variables with skewed distribution. For all baseline data, analysis of relationships between continuous variables was carried out using the Spearman correlation test for bivariate analyses, and non-parametric tests for comparing distributions. For the pilot supplementation study, two sets of patient samples were obtained at baseline with a one-month interval between samples. A further treatment sample was obtained after one month of vitamin D therapy so that each patient served as his/her own control. The first sample obtained at baseline served as control for comparison between the two baseline samples, and also for comparison with the post-treatment sample. The effects of supplemental vitamin D on genes of interest as well as peritoneal and circulating biomarkers were then analysed by using non-parametric tests for matched data. As *CAMP* is expressed by both macrophages (CD45^+^ and CD14^+^) and neutrophils (CD45^+^ and CD14^−^), the effects of supplemental vitamin D on *CAMP* mRNA expression and peritoneal hCAP concentrations were analysed by using a mixed linear model, which is a hierarchical method based on measurements (samples) made over time and on clusters of related statistical units (i.e. patients). This takes into account that samples over time from the same patient are not independent, and adjusts on variables that vary at the sample level (e.g. CD14/CD45 expression) or at the patient level (e.g. age). The impact of vitamin D treatment on *CAMP* mRNA expression and peritoneal hCAP concentration respectively were thus analysed, adjusted on CD14^−^/CD45^+^ expression, and age. The model results in coefficients (95% CI) estimating the impact of supplemental vitamin D adjusted on individual group-level co-variables, and taking into account the dependence of samples from a same patient. This model is a generalized linear multilevel model, so no linearity or normality assumptions were made. All statistical tests were performed at the two-sided 0.05 level of significance while an alpha of 0.10 was considered significant in multiple regression analyses. Analyses were performed using SPSS software 19.0 (SPSS Inc., Chicago) for Windows, and Stata 11/SE software (Statacorp LP, Texas).

## Supporting Information

S1 File **Supporting tables.** Table S1, Underlying primary renal disease leading for each patient in the baseline data and pilot study. Table S2, General linear mixed modelling for cathelicidin (*CAMP*) mRNA expression. In all models, samples were clustered within patients (ie. considered as a group-level variable). The glm Stata procedure was used, modelling with random effects. Results were presented as *coefficient (95% CI); p-value* only if significant according to the model run. Each line corresponds to a separate modelling, All genes expression data were tested separately, with adjustment variables defined in the 1^st^ column. *No adjustment on paediatric state for this model. The statistical analyses were made with raw ΔCt of mRNA expression; since a greater ΔCt corresponds to a decreased mRNA expression, a negative result corresponds to an increased expression of the gene of interest. Table S3, General linear Mixed modelling for cathelicidin protein (hCAP) concentration in the peritoneal dialysis effluent. In all models, samples were clustered within patients (ie. considered as a group-level variable). The glm Stata procedure was used, modelling with random effects. Results were presented as *coefficient (95% CI); p-value*. Each line corresponds to a separate modelling, and adjustment variables are defined in the 1^st^ column. *No adjustment on paediatric state for this model. Table S4, Effect of residual renal function at baseline. Correlation analyses were carried out using data for baseline sample 1, and analysed using Mann-Whitney-Wilcoxon tests.
(DOCX)

S2 File **Supporting figures.** Figure S1, Expression of vitamin D-related genes relative to CD14 and CD45 expression by cells isolated from peritoneal dialysate effluent. Correlation between mRNA expression for: A&B) *CYP27B1*; C&D) *VDR*; E&F) *CYP24A1* and percentage of PD cells that are non-monocytic (CD14^−^) (A, C, E) or monocytic (CD14^+^) (B, D, F). PD cells were isolated from CKD patient dialysate effluents from n = 40 baseline samples from 27 different patients (pre-vitamin D supplementation). Data for mRNA expression are shown as 1/ΔCt values. Figure S2, Correlation between concentrations of plasma fibroblast growth factor 23 (FGF23) and FGF23 in peritoneal dialysate effluent. Figure S3, Effect of therapeutic use of active calcitriol or vitamin D analog therapy on responses to vitamin D supplementation in the pilot study. Of the 12 patients who participated in the vitamin D supplementation pilot study, 8 were receiving therapy with active calcitriol or vitamin D analogs (analog therapy). A) serum concentrations of 25D (ng/ml) in patients at baseline 1 (B1), baseline 2 (B2) or following vitamin D supplementation (TT) according to use of analog therapy or no therapy. B) fold-suppression of hepcidin (*HAMP*) mRNA expression in peritoneal monocyte/macrophages following vitamin D supplementation (relative to cells from B1 samples), according to use of analog therapy or no therapy. ** = statistically different from analog therapy, p<0.01 (students t-test).
(DOCX)
