# Supplementary material for: Antibacterial Responses by Peritoneal Macrophages Are Enhanced Following Vitamin D Supplementation
Source: PLoS One. 2014 Dec 30;9(12):e116530. doi: 10.1371/journal.pone.0116530 (PMC4280222; doi:10.1371/journal.pone.0116530)
Supplement: S1 File — Supporting tables. Table S1, Underlying primary renal disease leading for each patient in the baseline data and pilot study. Table S2, General linear mixed modelling for cathelicidin (CAMP) mRNA expression. In all models, samples were clustered within patients (ie. considered as a group-level variable). The glm Stata procedure was used, modelling with random effects. Results were presented as coefficient (95% CI); p-value only if significant according to the model run. Each line corresponds to a separate modelling, All genes expression data were tested separately, with adjustment variables defined in the 1st column. *No adjustment on paediatric state for this model. The statistical analyses were made with raw ΔCt of mRNA expression; since a greater ΔCt corresponds to a decreased mRNA expression, a negative result corresponds to an increased expression of the gene of interest. Table S3, General linear Mixed modelling for cathelicidin protein (hCAP) concentration in the peritoneal dialysis effluent. In all models, samples were clustered within patients (ie. considered as a group-level variable). The glm Stata procedure was used, modelling with random effects. Results were presented as coefficient (95% CI); p-value. Each line corresponds to a separate modelling, and adjustment variables are defined in the 1st column. *No adjustment on paediatric state for this model. Table S4, Effect of residual renal function at baseline. Correlation analyses were carried out using data for baseline sample 1, and analysed using Mann-Whitney-Wilcoxon tests. (DOCX) [file pone.0116530.s001.docx]

| **Underlying disease** | **Number of patients** | **Vitamin D supplementation pilot study** |
| --- | --- | --- |
| Alport's Syndrome | 1 |  |
| ANCA + GN | 1 |  |
| Bilat. Cystic Dysplastia | 2 |  |
| Congenital Nephrotic Syndrome | 1 |  |
| FSGS | 2 | 2 |
| Glomerulonephritis | 1 | 1 |
| Glomerulonephritis Anca + | 1 | 1 |
| IgM Nephropathy | 1 |  |
| Immune Complex GN | 1 |  |
| MPGN Type I | 1 |  |
| Obstructive Uropathy, PUV | 1 | 1 |
| Pauci immnue GN | 1 |  |
| Reflux Nephropathy | 1 | 1 |
| Renal Dysplasia | 2 | 2 |
| RPKD | 1 |  |
| Unknown | 9 | 4 |

**Table S1. Underlying primary renal disease leading for each patient in the baseline data and pilot study.**

| **Modelling** | **Results** | |
| --- | --- | --- |
| *The 1^st^ baseline vs. after treatment* | |  |
| No adjustment | | NS |
| Adjusted on CD14-CD45+ | | CAMP: -0.87 (-1.67 to -0.07); p=0.03 |
| Adjusted on CD14+CD45+ | | NS |
| Adjusted on both (CD45+CD14+/-) | | NS |
| Adjusted on both and pediatric state | | NS |
| Adjusted on both and age | | NS |
| Adjusted on CD14-CD45+ and paediatric state | | CAMP: -0.89 (-1.59 to -0.19); p=0.01 |
| Adjusted on CD14-CD45+ and age | | CAMP: -0.90 (-1.68 to -0.12); p=0.02 |
| Adjusted on CD14-CD45+ and paediatric state and PTH | | CAMP: -0.80 (-1.56 to -0.03); p=0.04 |
| Adjusted on CD14-CD45+ and paediatric state and circulating FGF23 | | CAMP: -1.15 (-1.70 to -0.59); p<0.0001 |
| Adjusted on CD14-CD45+ and paediatric state and circulating FGF23 and PTH | | CAMP: -1.08 (-1.74 to -0.43); p=0.001 |

**Table S2. General linear mixed modelling for cathelicidin (*CAMP*) mRNA expression.**

In all models, samples were clustered within patients (ie. considered as a group-level variable). The glm Stata procedure was used, modelling with random effects. Results were presented as *coefficient (95% CI); p-value* only if significant according to the model run. Each line corresponds to a separate modelling, All genes expression data were tested separately, with adjustment variables defined in the 1^st^ column. ^*^No adjustment on paediatric state for this model. The statistical analyses were made with raw ΔCt of mRNA expression; since a greater ΔCt corresponds to a decreased mRNA expression, a negative result corresponds to an increased expression of the gene of interest.

| **Modelling** | **Results** |
| --- | --- |
| *The 1^st^ baseline vs. after treatment* |  |
| No adjustment | 356 (75 to 636); p=0.01 |
| Adjusted on CD14-CD45+ | 779 (140 to 1406); p=0.02 |
| Adjusted on CD14+CD45+ | 337 (51 to 624); p=0.02 |
| Adjusted on both (CD45+CD14+/-) | NS |
| Adjusted on both and pediatric state | NS |
| Adjusted on both and age | NS |
| Adjusted on CD14-CD45+ and paediatrics state | 779 (140 to 1406); p=0.02^*^ |
| Adjusted on CD14-CD45+ and age | 846 (288 to 1403); p=0.003 |
| Adjusted on CD14-CD45+ and paediatric state and PTH | 744 (98 to 1391); p=0.02 |
| Adjusted on CD14-CD45+ and paediatric state and circulating FGF23 | NS |
| Adjusted on CD14-CD45+ and paediatric state and circulating FGF23 and PTH | NS |

**Table S3. General linear Mixed modelling for cathelicidin protein (hCAP) concentration in the peritoneal dialysis effluent.**

In all models, samples were clustered within patients (ie. considered as a group-level variable). The glm Stata procedure was used, modelling with random effects. Results were presented as *coefficient (95% CI); p-value*. Each line corresponds to a separate modelling, and adjustment variables are defined in the 1^st^ column. ^*^No adjustment on paediatric state for this model.

| **Test** | | | | | | | | | | | | | | | | | | |
| --- | --- | --- | --- | --- | --- | --- | --- | --- | --- | --- | --- | --- | --- | --- | --- | --- | --- | --- |
|  | **Age** | **Vintage** | **KT/V** | **%CD14-45+** | **%CD14+45-** | **%double +** | **%double neg** | **CYP24A1** | **CYP27B1** | **VDR** | **CAMP** | **HAMP** | **PD-CAMP** | **Blood-FGF** | **PD-FGF** | **CR** | **BUN** | **25D** |
| U Mann-Whitney | 26.000 | 22.500 | 62.000 | 45.000 | 37.000 | 39.000 | 36.000 | 36.000 | 69.500 | 75.000 | 34.000 | 68.000 | 68.000 | 16.000 | 48.000 | 20.000 | 61.000 | 68.000 |
| W Wilcoxon | 197.000 | 212.500 | 98.000 | 216.000 | 52.000 | 54.000 | 207.000 | 72.000 | 259.500 | 265.000 | 205.000 | 258.000 | 258.000 | 169.000 | 201.000 | 173.000 | 97.000 | 104.000 |
| Z | -2.556 | -2.544 | -.556 | .000 | -.597 | -.447 | -.671 | -2.124 | -.345 | -.053 | -2.111 | -.425 | -.425 | -2.763 | -1.165 | -2.796 | -.408 | -.426 |
| Significance | .011 | .011 | .578 | 1.000 | .551 | .655 | .502 | .034 | .730 | .958 | .035 | .671 | .671 | .006 | .244 | .005 | .683 | .670 |

**Table S4.** Effect of residual renal function at baseline. Correlation analyses were carried out using data for baseline sample 1, and analysed using Mann-Whitney-Wilcoxon tests.
