# Supplementary material for: Antibacterial Responses by Peritoneal Macrophages Are Enhanced Following Vitamin D Supplementation
Source: PLoS One. 2014 Dec 30;9(12):e116530. doi: 10.1371/journal.pone.0116530 (PMC4280222; doi:10.1371/journal.pone.0116530)
Supplement: S2 File — Supporting figures. Figure S1, Expression of vitamin D-related genes relative to CD14 and CD45 expression by cells isolated from peritoneal dialysate effluent. Correlation between mRNA expression for: A&B) CYP27B1; C&D) VDR; E&F) CYP24A1 and percentage of PD cells that are non-monocytic (CD14−) (A, C, E) or monocytic (CD14+) (B, D, F). PD cells were isolated from CKD patient dialysate effluents from n = 40 baseline samples from 27 different patients (pre-vitamin D supplementation). Data for mRNA expression are shown as 1/ΔCt values. Figure S2, Correlation between concentrations of plasma fibroblast growth factor 23 (FGF23) and FGF23 in peritoneal dialysate effluent. Figure S3, Effect of therapeutic use of active calcitriol or vitamin D analog therapy on responses to vitamin D supplementation in the pilot study. Of the 12 patients who participated in the vitamin D supplementation pilot study, 8 were receiving therapy with active calcitriol or vitamin D analogs (analog therapy). A) serum concentrations of 25D (ng/ml) in patients at baseline 1 (B1), baseline 2 (B2) or following vitamin D supplementation (TT) according to use of analog therapy or no therapy. B) fold-suppression of hepcidin (HAMP) mRNA expression in peritoneal monocyte/macrophages following vitamin D supplementation (relative to cells from B1 samples), according to use of analog therapy or no therapy. ** = statistically different from analog therapy, p<0.01 (students t-test). (DOCX) [file pone.0116530.s002.docx]

**Figure S1. Expression of vitamin D-related genes relative to CD14 and CD45 expression by cells isolated from peritoneal dialysate effluent.**

Correlation between mRNA expression for: A&B) *CYP27B1*; C&D) *VDR*; E&F) *CYP24A1* and percentage of PD cells that are non-monocytic (CD14^-^) (A, C, E) or monocytic (CD14^+^) (B, D, F). PD cells were isolated from CKD patient dialysate effluents from n= 40 baseline samples from 27 different patients (pre-vitamin D supplementation). Data for mRNA expression are shown as 1/ΔCt values.

**Figure S2. Correlation between concentrations of plasma fibroblast growth factor 23 (FGF23) and FGF23 in peritoneal dialysate effluent.**

**Figure S3. Effect of therapeutic use of active calcitriol or vitamin D analog therapy on responses to vitamin D supplementation in the pilot study.**

Of the 12 patients who participated in the vitamin D supplementation pilot study, 8 were receiving therapy with active calcitriol or vitamin D analogs (analog therapy). A) serum concentrations of 25D (ng/ml) in patients at baseline 1 (B1) , baseline 2 (B2) or following vitamin D supplementation (TT) according to use of analog therapy or no therapy. B) fold-suppression of hepcidin (*HAMP*) mRNA expression in peritoneal monocyte/macrophages following vitamin D supplementation (relative to cells from B1 samples), according to use of analog therapy or no therapy. ** = statistically different from analog therapy, p<0.01 (students t-test).
